# Digital Health for Precision Prevention

**DOI:** 10.1055/s-0044-1800712

**Published:** 2025-04-08

**Authors:** Fleur Mougin, Kate Fultz Hollis, Lina F. Soualmia

**Affiliations:** 1 Univ. Bordeaux, Inserm, U1219, BPH, AHeaD team, Bordeaux, France; 2 Oregon Health & Science University Department of Biomedical Informatics and Clinical Epidemiology, Portland, Oregon, USA; 3 Univ. Rouen Normandie, Normandie Univ, LITIS UR 4108, F-76000 Rouen

**Keywords:** Medical Informatics, Health Information Technology, Digital Health, Precision Prevention, IMIA Yearbook of Medical Informatics

## Abstract

**Objectives** : To introduce the 2024 International Medical Informatics Association (IMIA) Year-book by the editors.

**Methods** : The editorial provides an introduction and overview to the 2024 IMIA Yearbook with the special theme, “Digital Health for Precision in Prevention”. The special topic, the survey papers and some of the best papers selected this year by section editors are introduced. Changes in the Yearbook editorial board are also described.

**Results** : IMIA Yearbook 2024 provides many perspectives on the popular topic called “Digital Health for Precision in Prevention”. The theme expresses the aim to provide the right intervention at the right time, adapted to the needs of each individual. Many sections presented original work on this year's theme, and all sections described notable contributions from 2023 in the various medical informatics specialties covered by the Yearbook.

**Conclusions** : The theme of “Digital Health for Precision in Prevention” is very important now when the rapid and extensive variety of digital tools grow exponentially.

## 1. Precision Prevention and Medical Informatics

Most health funding is allocated to patient treatment and care, even though investing in preventive measures would yield better outcomes [ 1 ]. Medical informatics has made health data readily accessible in digital form, simplifying its use. Additionally, digital tools enable the collection of supplementary data beyond standard healthcare contexts, providing a holistic view of individuals' health. This wealth of data creates unique opportunities to develop prevention policies aimed at groups with similar characteristics. The International Medical Informatics Association (IMIA) Yearbook editorial board has thus selected “Digital Health for Precision in Prevention” as the special topic, aiming to review recent advancements in this promising field.

For this special section, Gallego *et al.* [ 2 ] wrote a survey paper entitled “Precision Prevention: Using data to target the right intervention at the right intensity in the right community at the right time”. The authors first set out to define the complex notion of precision prevention combining definitions previously given in the literature. According to Gallego *et al.* , precision prevention is “ *the use of biologic, behavioral, socioeconomic, and epidemiologic data to inform the right intervention at the right intensity in the right community at the right time, in order to prevent or reduce illness and improve health* ”. The authors then present the different types of data that can be used in this context, with particular emphasis on the social determinants of health (SDOH), whose use is now recognized as essential, especially in the wake of the Covid-19 pandemic. After describing the HL7 Gravity Project, which aims to standardize SDOH data, Gallego *et al.* illustrate how these data have been used in recent work for precision prevention purposes.

The editors of the special section, Brian Dixon and John Holmes, detail in their synopsis the eleven papers they had selected as candidate best papers [ 3 ]. They note applications in the field of precision nutrition, precision medicine, and precision public health.

The survey paper from the Public Health and Epidemiology Informatics (PHEI) is also related to precision prevention [ 4 ]. He *et al.* reviewed 58 articles and Web sites related to “digital informatics”, “precision in prevention”, “precision epidemiology”, “public health surveillance”, and “clinicogenomics”. They describe digital solutions in precision epidemiology, illustrated by their use in public health surveillance and in the management of COVID-19 and chronic obstructive pulmonary disease in particular. The two best papers presented by Georgeta Bordea, Gayo Diallo, and Cécilia Samieri in their synopsis of the PHEI section [ 5 ] show: (i) the value of using large language models on unstructured electronic medical record data to improve clinical and operational predictive tasks, and (ii) an effective increase in short-term physical activity through personalized digital health interventions.

Sulieman *et al.* [ 6 ] present in the Decision Support (DS) section a scoping review of precision medicine particularly in clinical decision support (CDS) and found that pharmacogenomics is the most implemented precision medicine intervention based on published studies. For the DS review of the year, DS editors Christoph Lehmann and Vignesh Subbian added some interesting papers and themes. Their synopsis [ 7 ] highlighted the application of CDS in environments ranging from primary care to pediatric intensive care units, and even spaceflight, and addressing conditions such as acute kidney injury and bronchiolitis.

The Consumer Health Informatics (CHI) section also focused on how AI can enhance efforts to improve precision and security of health surveillance systems. CHI editors Annie Lau and Pascal Staccini present a survey paper by Canfell *et al.* [ 8 ] who conclude that CHI and precision prevention represent a potential future vanguard in shifting from traditional and inefficient break-fix to predict-prevent models of healthcare. Meaningful researcher, practitioner, and consumer partnerships must focus on generating high-quality evidence from methodologically robust study designs to support consumer health informatics as a core enabler of precision prevention.

## 2. Other highlights of the 33 ^rd^ Edition of the IMIA Yearbook

Although the theme “Digital Health for Precision in Prevention” of the 33 ^rd^ edition of the IMIA Yearbook is a hot topic, some sections have not selected articles on this topical subject. However, many of the section surveys focused on new perspectives and tools in artificial intelligence (AI) and machine learning. In the Bioinformatics and Translational Informatics (BTI) section [ 9 ] Long *et al.* The Signals, Sensors, and Imaging Informatics section (SSII) survey paper [ 10 ] provides a review on computer-aided design systems for automated Alzheimer's disease detection, focusing on different data types: signals and sensors, medical imaging, and electronic medical records. In their synopsis [ 11 ], SSII editors Leticia Rittner, Christian Baumgartner, and Thomas M. Deserno remark on advanced approaches focusing on deep learning-based image processing, analysis, and indicate a shift in the research field from sensor technology development to biosignal and image analysis.

A subject that we expected to find in the literature of 2023 is the use of large language models (LLMs) in the health domain. Indeed, the survey paper of the Knowledge and Representation Management (KRM) and the Natural Language Processing (NLP) sections deal with this hot topic. Bona has carried out a review of 15 articles published in 2022 or 2023 on KRM related to long COVID and the combination of KRM approaches with generative LLMs [ 12 ]. One the one hand, the author shows that KRM – often combined with machine or deep learning methods – is useful for understanding the clinical manifestations of long COVID and its similarities to other conditions. On the other hand, Bona highlights that KRM can be helpful in improving LLM results (and limits hallucinations, in particular) and LLMs in enhancing gene set analysis and interfacing between humans and knowledge represented in ontologies. Sarker *et al.* give a broad overview of how LLMs have revolutionized biomedical natural language processing [ 13 ]. The authors first point out the need to train LLMs on medical texts and categorize medical LLMs that have been developed recently according to multiple characteristics. Sarker *et al.* then describe the different types of data (medical literature, electronic health records, social media) and the NLP tasks on which medical LLMs have been used. Finally, they conclude on the limitations, challenges and risks that LLMs still face.

The Cancer Informatics survey [ 14 ] this year focused on relatively new digital medicine: cancer care and treatment with AI and large language model (LLM) tools. Benson *et al.* note that “ *(t)hese technologies hold the potential to transform clinical care by reducing burdensome clinical workloads, improving patient and caregiver understanding of cancer, and supporting scientific discovery and clinical advances. However, at this point in time, more foundational work on the capabilities, limitations, and evaluation approaches of these technologies is needed* ”.

The survey paper of the Clinical Information System (CIS) section by Peek *et al.* [ 15 ]provides a comprehensive analysis of the deployment of clinical decision support systems (CDSS) based on machine learning methods in clinical practice. In particular, Peek *et al.* highlighted the need to use theories and frameworks from implementation science and to involve stakeholders other than healthcare professionals. A comparison with rule-based CDSS is also presented, highlighting similarities in terms of usability of the system and additional workload but also some differences, particularly with regard to confidence and transparency.

In the survey paper of the Health Information Exchange (HIE) section, Dullabh *et al.* [ 16 ] review the literature on existing HIE initiatives, standards and technology solutions for the reuse of patient-centered data not generated in the course of care. Despite the progress made, challenges remain in integrating multiple data sources, ensuring widespread adoption of health information technologies and addressing privacy concerns, which require public policies and additional standards.

We are very pleased to welcome back Christel Daniel (former IMIA section editor) and Peter Embi to do a comprehensive review [ 17 ] of clinical research informatics (CRI) themes in the last decade. Daniel and Embi leveraged two annual review processes, the “AMIA CRI year-in-review” and IMIA yearbook approach, and ultimately selected 205 impactful papers to analyze in-depth important informatics issues we have in clinical research.

Finally, the Human Factors and Organizational Issues (HFOI) section focuses on how the latest systems to improve health care can be improved for the users. The survey paper [ 18 ] this year has extensive reviews on a variety of HFOI topics. Kushniruk and Kaufman “ *examine a range of phenomena from micro-level issues affecting individual technology users to meso-level issues impacting healthcare teams and institutions. This has included continued work in areas such as participatory design of systems, improved user requirements modelling and specification, improved methods for ensuring healthcare system safety, and integration of new applications such as AI tools and systems into healthcare in ways that do not interfere with workflow and that can augment and extend healthcare work* ”.

## 3. Changes in the Yearbook Editorial Team

This year was a very significant one for the Yearbook editorial board, as we were so sad to lose one of our key members: Eta S Berner, who had been Professor of Medical Informatics and section editor of HIE (formerly Health Information Management) since 2017. Eta was not only an excellent teacher and inspiring mentor, but she also made major research contributions to the field of clinical decision support systems, as recently highlighted by Bakken *et al.* [ 19 ].

We are very grateful to Sue S. Feldman, Professor at the School of Health Professions, University of Alabama at Birmingham (Alabama, United States), for stepping in to help replace Eta as editor of the HIE section. For the Cancer Informatics section, we welcome two new editors: Sanjay Aneja from the Yale Cancer Center, the Department of Therapeutic Radiology, and the Department of Bioinformatics and Data Science, Yale School of Medicine, USA and Ravi B. Parikh from the Abramson Cancer Center, Department of Medicine, and Department of Medical Ethics and Health Policy at the University of Pennsylvania, USA. We are happy to continue to have this recent section of the Yearbook in very capable hands.

On the departure side, we want to thank Jean Charlet so much as he has been editor of KRM since 2013 and has been committed to the success and quality of the Yearbook. We would also like to thank Georgeta Bordea and Gayo Diallo, editors of the PHEI section, for their valuable contribution since 2021, as well as Cécilia Samieri, who has given them a helping hand over the past two years.

Finally, we are delighted to welcome Yann Rousselot to the editorial board, succeeding Martina Hutter, who has even been involved with the Yearbook for the 25 ^th^ year, to ensure the best possible transition with Yann.

## References

[1] Canfell OJ, Littlewood R, Burton-Jones A, Sullivan C. Digital health and precision prevention: shifting from disease-centred care to consumer-centred health. Aust Health Rev. 2022 Jun;46(3):279-283. doi: 10.1071/AH21063. 10.1071/AH21063 34882538

[2] Gallego E, Hinz EM, Massey B, Tilson EC, Tenenbaum JD. Precision Prevention: Using data to target the right intervention at the right intensity in the right community at the right time. Yearb Med Inform. 2024;6-17. doi: 10.1055/s-0044-1800713 10.1055/s-0044-1800713 PMC12020636 40199283

[3] Dixon BE, Holmes JH. Special Section on Digital Health for Precision Prevention: Notable Papers that Leverage Informatics Approaches to Support Precision Prevention Efforts in Health Systems. Yearb Med Inform. 2024;70-72. doi: 10.1055/s-0044-1800721 10.1055/s-0044-1800721 PMC12020638 40199291

[4] He Q, Silva PJ, Ory M, Wang N, Ramos KS. Application of Digital Informatics in Precision Prevention, Epidemiology, and clinicogenomics Research to Advance Precision Healthcare. Yearb Med Inform. 2024;250-261. doi: 10.1055/s-0044-1800753 10.1055/s-0044-1800753 PMC12020529 40199312

[5] Diallo G, Bordea G, Samieri C. Exploring the Latest Advances in Public Health and Epidemiology Informatics. Yearb Med Inform. 2024;262-264. doi: 10.1055/s-0044-1800754 10.1055/s-0044-1800754 PMC12020530 40199313

[6] Sulieman L, McCoy AB, Samal L, Peterson JF. The use of Precision Medicine to support the precision of Clinical Decisions in care delivery. Yearb Med Inform. 2024;168-174. doi: 10.1055/s-0044-1800738 10.1055/s-0044-1800738 PMC12020522 40199302

[7] Lehmann C and Subbian V. Advances in Clinical Decision Support Systems:Contributions from the 2023 Literature. Yearb Med Inform. 2024;175-177. doi: 10.1055/s-0044-1800739 10.1055/s-0044-1800739 PMC12020640 40199303

[8] Canfell OJ, Woods L, Robins D, Sullivan C. Consumer Health Informatics to Advance Precision Prevention. Yearb Med Inform. 2024;149-157. doi: 10.1055/s-0044-1800735 10.1055/s-0044-1800735 PMC12020525 40199300

[9] Long Y, Novak L, Walsh CG. Searching for Value Sensitive Design in Applied Health AI: A Narrative Review. Yearb Med Inform. 2024;75-82. doi: 10.1055/s-0044-1800723 10.1055/s-0044-1800723 PMC12020519 40199292

[10] Dehghani F, Derafshi R, Lin J, Bayat S, Bento M. Alzheimer Disease Detection Studies: Perspective on Multi-Modal Data. Yearb Med Inform. 2024;266-276. doi: 10.1055/s-0044-1800756 10.1055/s-0044-1800756 PMC12020631 40199314

[11] Rittner L, Baumgartner C, Deserno TM. Sensors, Signals, and Imaging Informatics: Best contributions from 2023. Yearb Med Inform. 2024;277-279. doi: 10.1055/s-0044-1800757 10.1055/s-0044-1800757 PMC12020550 40199315

[12] Bona JP. Knowledge Representation and Management in the Age of Long Covid and Large Language Models: A 2022-2023 Survey. Yearb Med Inform. 2024;216-222. doi: 10.1055/s-0044-1800747 10.1055/s-0044-1800747 PMC12020515 40199308

[13] Sarker A, Zhang R, Wang Y, Xiao Y, Das S, Schutte D, et al. Natural Language Processing for Digital Health in the Era of Large Language Models. Yearb Med Inform. 2024;229-240. doi: 10.1055/s-0044-1800750 10.1055/s-0044-1800750 PMC12020548 40199310

[14] Benson R, Elia M, Hyams B, Chang JH, Hong JC. A Narrative Review on the Application of Large Language Models to Support Cancer Care and Research. Yearb Med Inform. 2024;90-98. doi: 10.1055/s-0044-1800726 10.1055/s-0044-1800726 PMC12020524 40199294

[15] Peek N, Capurro D, Rozova V, van de Veer S. Bridging the Gap: Challenges and Strategies for the Implementation of Artificial Intelligence-based Clinical Decision Support Systems in Clinical Practice. Yearb Med Inform. 2024;103-114. doi: 10.1055/s-0044-1800729 10.1055/s-0044-1800729 PMC12020628 40199296

[16] Dullabh P, Dhopeshwarkar R, Desai PJ. New Horizons for Consumer-Mediated Health Information Exchange. Yearb Med Inform. 2024;179-190. doi: 10.1055/s-0044-1800741 10.1055/s-0044-1800741 PMC12020518 40199304

[17] Daniel C and Embi P. Clinical Research Informatics: A Decade-in-Review. Yearb Med Inform. 2024;127-142. doi: 10.1055/s-0044-1800732 10.1055/s-0044-1800732 PMC12020646 40199298

[18] Kushniruk A and Kaufman D. Human Factors and Organizational Issues in Health Informatics: Review of Recent Developments and Advances. Yearb Med Inform. 2024;196-209. doi: 10.1055/s-0044-1800744 10.1055/s-0044-1800744 PMC12020533 40199306

[19] Bakken S, Cimino JJ, Feldman S, Lorenzi NM. Celebrating Eta Berner and her influence on biomedical and health informatics. J Am Med Inform Assoc. 2024 Feb 16;31(3):549-551. doi: 10.1093/jamia/ocae011 10.1093/jamia/ocae011 PMC10873777 38366906

